# Staphylococcal Scalded Skin Syndrome and Bullous Impetigo

**DOI:** 10.3390/medicina57111157

**Published:** 2021-10-24

**Authors:** Morgan Brazel, Anand Desai, Abhirup Are, Kiran Motaparthi

**Affiliations:** 1 University of Florida College of Medicine, Gainesville, FL 32610, USA; brazemor@ufl.edu (M.B.); acare2019@hotmail.com (A.A.); 2 Department of Dermatology, University of Florida College of Medicine, Gainesville, FL 32610, USA; adesai@dermatology.med.ufl.edu

**Keywords:** staphylococcal scalded skin syndrome, bullous impetigo, *Staphylococcus aureus*

## Abstract

Staphylococcal scalded skin syndrome (SSSS) and bullous impetigo are infections caused by *Staphylococcus aureus*. The pathogenesis of both conditions centers around exotoxin mediated cleavage of desmoglein-1, which results in intraepidermal desquamation. Bullous impetigo is due to the local release of these toxins and thus, often presents with localized skin findings, whereas SSSS is from the systemic spread of these toxins, resulting in a more generalized rash and severe presentation. Both conditions are treated with antibiotics that target *S. aureus.* These conditions can sometimes be confused with other conditions that result in superficial blistering; the distinguishing features are outlined below.

**Staphylococcal scalded skin syndrome**

## 1. Introduction

Staphylococcal scalded skin syndrome (SSSS), also known as Ritter disease, is a potentially life-threatening infection caused by certain strains of *Staphylococcus aureus* (*S. aureus*) that release exfoliative toxins. Clinically, it is characterized by denudation of the skin and presents as large superficial blisters [1]. The overall incidence of SSSS in the general population is estimated to be between 0.09 and 0.56 cases per one million people [2]. However, SSSS is most commonly seen in children under the age of six. A study in the Czech Republic reported an incidence as high as 250 per one million children less than one year old [3]. The increased incidence in young children is due to a lack of protective antibodies against exfoliative toxins and decreased renal clearance of the toxins as a result of immature renal function [4]. The mortality rate of SSSS is less than five percent in children and greater than sixty percent in adults, likely due to an underlying immunodeficiency or comorbidity [2,3,4].

## 2. Pathophysiology

*S. aureus* is a Gram-positive bacterium that frequently colonizes the eyes, ears, nares, umbilicus, and groin. All strains of *S. aureus* produce toxins, but only five percent of them release the exfoliative toxins A and B (ETA, ETB) that cause SSSS [2,5]. In a recent study by Vernali et al., fifty-nine patients diagnosed with SSSS were identified. Aerobic cultures identified methicillin-sensitive *S. aureus* (MSSA) in 37.3% of patients, methicillin-resistant *S. aureus* (MRSA) in 3.4% of patients, normal cutaneous flora in 15.3% of patients, a culture with no growth in 15.3% of patients, and 28.8% did not have a culture performed. Among the patients with a pathogen isolated on culture, 8.3% revealed MRSA, and 75% revealed resistance to clindamycin [6]. Of note, there are no descriptions of vancomycin-resistant *S. aureus* (VRSA) reported in the literature. Most commonly, *S. aureus* of phage group II types 3A, 3B, 3C, 55, and 71 are the strains that produce ETA and ETB and are, therefore, responsible for the sequelae of SSSS. ETA and ETB are serine proteases that are released at the initial site of infection before spreading hematogenously to the stratum granulosum of the epidermis [4]. Once there, the toxins accumulate in the skin and digest desmoglein-1, a desmosomal cadherin that maintains keratinocyte adhesion [7]. As a result, there is loss of keratinocyte cell-to-cell adhesion in the stratum granulosum, followed by denudation and the formation of superficial bullae [2]. Cellular adhesion in the lower epidermis and mucosal membranes is maintained in SSSS since desmoglein-3 is enriched in these sites. Desmoglein-3 is not lysed by ETA and ETB and is able to compensate for the destruction of desmoglein-1 [4].

## 3. Clinical Features in Children

SSSS is most commonly seen in children less than five years of age, with the majority of cases occurring between the ages of two and three [4]. Neonates in the first few weeks of life are relatively protected from SSSS, due to the presence of desmoglein 3 throughout the epidermis. In comparison, desmoglein 3 is only present in the deep epidermis of adult skin [8]. The relatively high amount of desmoglein 3 in neonatal skin is compensates for the loss of function of desmoglein 1 that occurs due to ETA and ETB, protectingneonates from SSSS [7]. However, by the end of the neonatal period and in infancy, children are at high risk, as the relative proportion of desmoglein 1 in the upper strata of the epidermis begins to transition toward that of adult skin.

**Figure 1:**
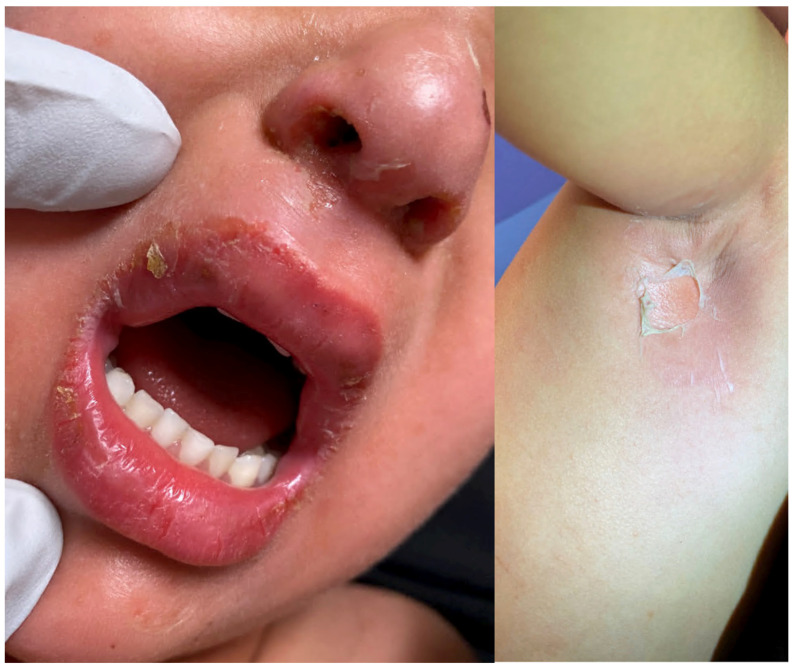
Staphylococcal scalded skin syndrome. Superficial blistering on the face (**left**) and in the axilla (**right**). Original image provided by Kiran Motaparthi, MD.

SSSS usually begins in children with a prodrome of irritability, generalized fatigue, and fever [1]. The most common sites of initial localized infection by *S. aureus* in children include the conjunctiva, nose, throat, diaper area, umbilical stump, and circumcision or other surgical wounds (Table 1) [2,4]. Within 24–48 h, tender erythematous patches develop that usually begin on the face and flexural regions, such as the axillae, groin, and neck (Figure 1). Several hours later, fragile blisters develop within the erythematous areas with accumulated fluid ranging from thin, sterile liquid to frank pus [3]. These blisters progress in size to form bullae which rupture easily and cause desquamation. The skin develops a classic wrinkled appearance, due to the formation of the flaccid bullae that is referred to as “sad man facies” [9]. Patients have a positive Nikolsky sign on physical exam, as gentle pressure on the affected skin will cause exfoliation of the upper epidermis [4]. There is usually a second period of desquamation in the next 10 days, and in most cases, the skin heals without scarring within two weeks [2,4]. The mortality rate in children is consistently less than 5% (Table 1), and poor outcomes are associated with sepsis, electrolyte imbalance, and dehydration [3,4]. A review from 2008 to 2012 found the inpatient mortality rate of children with SSSS in the United States to be as low as 0.33% [10].

## 4. Clinical Features in Adults

It is very rare for adults to develop SSSS, as they possess neutralizing antibodies against the exotoxins and also have increased renal clearance of the exotoxins [11]. In contrast to affected children, who are typically otherwise healthy, adults who develop SSSS are almost always immunocompromised due to comorbidities, such as severe renal disease, HIV infection, or malignant neoplasms [2]. The initial, localized infection by *S. aureus* in adults can result from an abscess, arteriovenous fistula infection, pneumonia, osteoarthritis, or septic arthritis (Table 1) [2,3]. The clinical presentation is similar to that in children with fever, erythematous patches, bullae formation, and desquamation [2]. In contrast to pediatric patients, the mortality rate of SSSS is greater than 60% in adults, due to predisposing comorbid conditions (Table 1) [3,4].

## 5. Diagnostic Workup

While the diagnosis of SSSS is mainly clinical, there are several tests that can be performed for confirmation. Since the causative exfoliative toxins spread hematogenously to the skin from a distant primary site of infection, *S. aureus* itself is not found in the skin lesions and obtaining cultures of the bullae or erosions in SSSS is of little utility [4]. Blood cultures are typically negative in the pediatric population. The majority of adults, however, will have bacteremia due to *S. aureus*, resulting from increased severity and underlying comorbidities [2]. *S. aureus* can also be cultured from the site of primary infection, such as the umbilicus, nasopharynx, conjunctiva, or surgical wounds in both children and adults [5]. Nasopharyngeal and periorificial cultures (perianal, perioral, periocular, nasal) are most likely to be positive [1].

**Figure 2:**
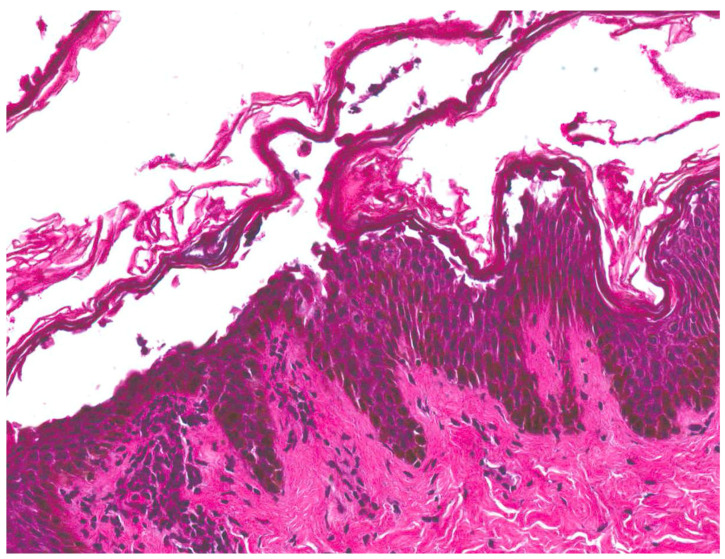
Staphylococcal scalded skin syndrome. Subcorneal acantholysis without inflammation, crust, or bacterial cocci (H&E, 200× magnification). Original image provided by Kiran Motaparthi, MD.

Although not typically necessary, a skin biopsy can help support a diagnosis of SSSS [5]. Biopsy, including frozen sections, reveals superficial intraepidermal cleavage beneath the stratum corneum (Figure 2) and can help to differentiate SSSS from other blistering conditions in the acute setting. For example, SSSS is rapidly differentiated from Stevens–Johnson syndrome and toxic epidermal necrolysis, given that these disorders demonstrate a subepidermal cleft and prominent keratinocyte necrosis. [2].

## 6. Differential Diagnosis

The differential diagnosis for SSSS includes a variety of often diffuse blistering disorders. Carefully differentiating between various blistering diseases is essential to guiding treatment. While these disorders can often be distinguished clinically, biopsy can also help differentiate in several cases. The table below compares and contrasts various diseases that can be confused with SSSS (Table 2).

## 7. Management

SSSS should be treated early with penicillinase-resistant penicillins, such as nafcillin or oxacillin [2]. For children, 100–150 mg/kg per day of intravenous (IV) nafcillin or oxacillin should be given with doses divided every six hours. The maximum daily dose is 12 g per day. Alternatives include a first- or second-generation cephalosporin. Intravenous cefazolin can be given to children at 50–100 mg/kg per day divided into doses every eight hours. The maximum daily dose is 6 g per day for adults [21]. Nafcillin can be given at 2 g every four hours. Alternatively, 2 g of IV cefazolin can be given every eight hours [22]. Vancomycin should be used if the patient lives in an area with a high prevalence of MRSA, or if the patient does not respond to initial therapy [4]. For children, IV vancomycin 45 mg/kg per day divided into doses every eight hours should be given and should not exceed the maximum daily dose of 2 g per day [21]. In adults, vancomycin is typically dosed 1 g every twelve hours [22]. Antibiotic selection may also be determined by antibiotic sensitivities if available. In mild cases, the patient can take oral antibiotics as an outpatient. However, in more severe cases, patients will require inpatient hospitalization and IV antibiotics [3]. A patient who is initially on IV antibiotics can be switched to oral antibiotics when they begin to improve clinically and if they are able to swallow. Recommended oral antibiotic regimens include cephalexin 500 mg every six hours, dicloxacillin 500 mg every six hours, and trimethoprim/sulfamethoxazole 160 mg/800 mg every twelve hours [22]. The total duration of antibiotic treatment is 10 days, but treatment can be lengthened if needed. In the past, clindamycin was frequently added to the antibiotic treatment regimen, given its ability to inhibit the production of bacterial toxins [1]. However, newer data published by Wang et al. found that SSSS-associated strains of staphylococci demonstrate higher rates of resistance to clindamycin than other types of staphylococci. Sixty-three percent of SSSS-associated isolates of *S. aureus* demonstrated resistance to clindamycin, and there was no benefit observed in patients with SSSS who were treated with clindamycin, compared to those treated without clindamycin [23].

Supportive care with IV fluids may be indicated to prevent dehydration [23]. Pediatric patients may receive a bolus of fresh frozen plasma (FFP) that is 10 percent of their circulating volume followed by maintenance hydration in order to compensate for lost fluids. If a child does not benefit from FFP, intravenous immunoglobulin (IVIg) can be given as a third-line treatment. IVIg should be administered over five days (0.4 g/kg per day) to neutralize the pathogenic exotoxins [2]. Other supportive measures, such as wound care and pain management, may also be employed. Sterile, saline-soaked gauze is often applied to denuded areas with a layer of emollients to reduce tenderness. Topical antibiotics, such as mupirocin and fusidic acid, can also be used at the affected site and at the site of original colonization for decolonization [4,5,6]. Acetaminophen and opiates may be administered for pain control. However, non-steroidal anti-inflammatories (NSAIDS) can impair kidney function and should therefore be avoided in patients with SSSS [4].

Recurrence of SSSS is extremely rare with only a handful of cases reported in the literature. Relapse can be prevented with proper hygiene measures, such as barrier techniques, handwashing, and proper cleaning of equipment [24].

**Bullous Impetigo**

## 1. Introduction

**Figure 3:**
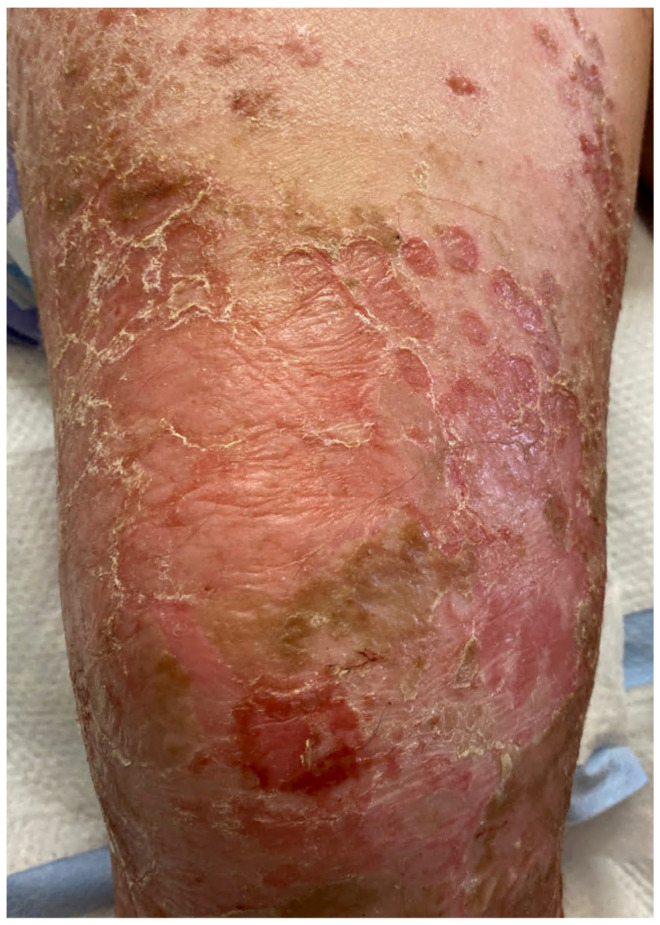
Bullous impetigo. After bullae rupture, superficial erosions and surrounding collarettes of scale remain, along with a thin brown crust. Original image provided by Kiran Motaparthi, MD.

Impetigo is the most common bacterial infection in children [25]. It is a highly contagious infection of the skin that affects the superficial layers of the epidermis [26]. The majority of cases of impetigo (70 percent) are classified as nonbullous, while bullous impetigo accounts for the remaining 30 percent of cases [27]. Bullous impetigo is almost exclusively caused by *S. aureus*. It is characterized by small vesicles that progress to form flaccid bullae [26]. These bullae can rupture very easily, which leaves behind collarettes of scale and a thin brown crust (Figure 3) [27]. In contrast to nonbullous impetigo, bullous impetigo does not form a honey-colored crust. Bullous impetigo is mostly commonly seen in infants and children, and 90% of cases occur in children less than 2 years old [26].

## 2. Pathophysiology

Primary impetigo is due to direct bacterial invasion of normal skin. Alternatively, secondary impetigo occurs when a previous wound site or an area of compromised skin becomes infected [26]. While nonbullous impetigo may be caused by either *Staphylococcus aureus*, *Streptococcus pyogenes*, or a combination of the two, bullous impetigo is exclusively caused by exfoliative toxin-producing strains of methicillin-sensitive or methicillin-resistant *Staphylococcus aureus* [27]. The exfoliative toxins A, B, and D, produced locally by these strains of *Staphylococcus aureus*, cleave desmoglein-1. This results in loss of keratinocyte adhesion in the granular layer of the epidermis [28]. These exfoliative toxins are the same toxins that are involved in SSSS. However, in SSSS, the toxins spread hematogenously from a distant site and produce systemic symptoms. In contrast, the toxins are localized to the sites of skin infection in bullous impetigo, due to the presence of lesional bacteria [27].

## 3. Clinical Features and Diagnostic Workup

The lesions of bullous impetigo are usually found on the trunk, extremities, and intertriginous areas, such as the axillae, neck folds, and diaper area [26,27]. They appear in well-demarcated clusters without any surrounding erythema or edema [28]. They begin as small vesicles, which rapidly progress to superficial flaccid bullae containing clear or yellow fluid that becomes dark or purulent. Often, the blisters rupture, resulting in red annular erosions and a rim of scale. This differs from SSSS, which initially presents as diffuse erythema with flexural prominence that over time can progress to flaccid bullae with superficial desquamation. The perioral area is often involved. Of note, lesional skin in bullous impetigo will contain colonies of *Staphylococcus aureus*. In contrast, the blisters and erosions of SSSS are sterile [28]. Associated regional lymphadenopathy is rare [25].

**Figure 4:**
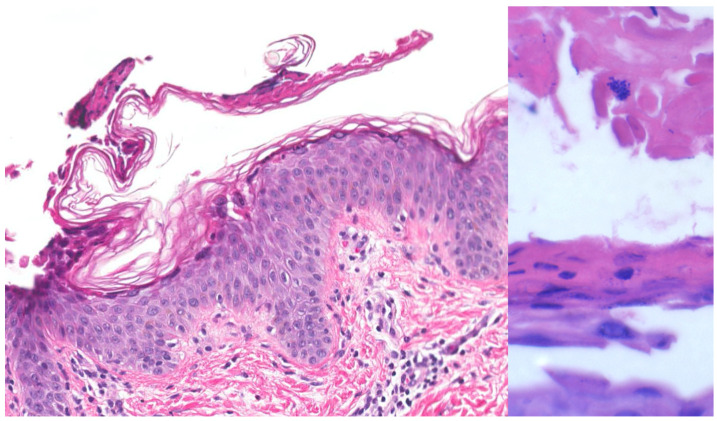
Bullous impetigo. Subcorneal acantholysis (left, H&E, 200x magnification) and cocci in clusters within the stratum corneum (right, H&E, 600× magnification). Original image provided by Kiran Motaparthi, MD.

History and clinical presentation are usually sufficient to make the diagnosis of bullous impetigo. However, bacterial cultures can be used to confirm the diagnosis and should also be obtained if resistant strains, such as methicillin-resistant *Staphylococcus aureus*, are suspected. A skin biopsy (Figure 4) can be performed if the case is not responsive to standard treatment [26].

While complications may arise, impetigo is generally a self-limited condition [27]. The infection heals in 14–21 days without treatment and within 10 days with treatment [26]. Bullous impetigo can rarely progress to SSSS, which presents with systemic symptoms, such a fever, malaise, and poor feeding [27].

## 4. Differential Diagnosis

The differential diagnosis for bullous impetigo includes conditions that present with annular scaly or crusted plaques, or superficial ulcerations. Carefully differentiating between these conditions is essential to guiding treatment. Often, these disorders can be distinguished clinically; however, biopsy can also help differentiate in several cases. The table below compares and contrasts dermatoses that are sometimes confused with bullous impetigo (Table 3).

## 5. Management

Treatment for bullous impetigo is based on disease severity. For limited disease due to MSSA or MRSA, first line treatment is typically topical mupirocin. Other topical options include retapamulin or ozenoaxcin. One study showed that 3–4 days of ozenoxacin resulted in 75% pathogen clearance, versus retapamulin, which demonstrated 60% pathogen clearance over the same time frame [30]. If disease is extensive (more than 5 lesions), resistant to topical therapy, or has associated complications such as cellulitis, systemic antibiotics can be pursued. For MSSA, cephalexin or dicloxacillin are first-line treatments. Second-line treatments for extensive disease include oral erythromycin or clarithromycin. Coverage for MRSA is indicated in MRSA endemic areas or based on cultures [26]. For extensive impetigo due to MRSA, first-line treatment options include doxycycline or clindamycin. Trimethoprim–sulfamethoxazole also provides coverage for MRSA but will not provide coverage for streptococcal disease.

Fluid from lesions can be cultured to support diagnosis and facilitate antibiotic selection. Definitive evidence to support bacterial culture is lacking. Nonetheless, several indications performing cultures are proposed, including the identification of MRSA prior to narrowing antimicrobial coverage [26,27]. Cultures may also be indicated in refractory cases. If patients develop multiple episodes of impetigo, decolonization can be considered. This involves applying mupirocin to the nares for 5 days each month. Chlorhexidine rinses or dilute bleach baths can be used concomitantly with mupirocin to improve the effectiveness of decolonization.

## Figures and Tables

**Table 1:** Clinical features of children and adults with SSSS.

	Children	Adults
Site of primary infection	Conjunctiva, nose, throat, diaper area, umbilical stump, or circumcision wound	Abscess, arteriovenous fistula infection, pneumonia, osteoarthritis, or septic arthritis
Common comorbidities	None	Immunodeficiency and renal disease
Blood cultures	Negative	Positive
Mortality rate	<5%	>60%

**Table 2:** Differential diagnosis of SSSS.

Disorder	Demographics	Triggers	Clinical Presentation	Histopathology	Clinical Course
**Staphylococcal scalded skin syndrome (SSSS)** [1,2,3,4]	Most commonly seen in children under the age of six	Initial localized infection by *S. aureus*	Prodrome of irritability, generalized fatigue, and fever followed by the progression of skin lesions. Positive Nikolsky sign	Superficial intraepidermal cleavage beneath the stratum corneum	Within 24–48 h, tender erythematous patches develop. Several hours later, fragile blisters develop within the areas. The blisters progress to form bullae, which rupture easily and then desquamate.
**Stevens-Johnson syndrome(SJS)/Toxic epidermal necrolysis (TEN)** [12]	Most common in older children and adults	80% of cases are linked to medication intake: sulfa drugs, allopurinol, tetracyclines, anticonvulsants, and NSAIDS	Full thickness epidermal detachment often involving the trunk and proximal extremities; mucosal surfaces affected in >90% of cases. Lesions start as red to dusky macules that coalesce. The necrotic epidermis detaches from the dermis and results in bullae formation. Nikolsky and Asboe–Hansen signs are positive	Uninvolved stratum corneum, vacuolar interface dermatitis with necrotic keratinocytes in the epidermis, with progression to full thickness epidermal necrosis	Lesions appear 7–21 days after drug exposure. Progresses for about 4–5 days before entering a plateau phase. Complete healing can take up to several weeks. Mortality rate near 5% for SJS, but up to 50% in TEN
**Acute generalized exanthematous pustulosis** [13,14]	Can occur in any age group. More common in women	Most cases linked to medication intake: aminopenicillins, cephalosporins, clindamycin, macrolide, and calcium channel blockers	Small non-follicular pustules arising in areas of background erythema often initially on face and flexural sites. Mucosal involvement sometimes present. Associated with fever and facial edema	Subcorneal, and/or intraepidermal pustules with eosinophilic and neutrophilic infiltrates and papillary dermal edema	Lesions appear <4 days after drug exposure then heal quickly over a few days with no evidence of scarring as level of split is subcorneal
**Bullous impetigo** [4,15]	Newborns and infants most commonly affected	Caused by *S. aureus* production of exfoliative toxins which cleave DSG1 resulting in acantholysis and bullae formation	Small vesicles that grow into tense bullae that rupture and leave behind a narrow rim of scale. The bullae appear in well-demarcated clusters at the initial site of infection. Systemic involvement is rare. Nikolsky sign is negative and culture of bullae or erosions is positive	Loss of cell adhesion in the superficial epidermis (granular layer) resulting in a subcorneal blister; mixed dermal inflammatory infiltrate, crusting, epidermal hyperplasia, and lesional cocci in clusters	Usually resolves within 3–6 weeks. However, high risk patients may develop SSSS due to dissemination of exfoliative toxin
**Toxic shock syndrome (TSS)** [16,17]	Usually a result of vaginal or surgical wound colonization	Typically caused by *S. aureus* (produces toxic shock syndrome toxin-1) or *S. pyogenes*	Fever >102 F, desquamative rash, hypotension, involvement of 3 or more organ systems. Symptoms often include- myalgias, vomiting, diarrhea, headache, pharyngitis. Rash is less common when caused by *S. pyogenes*	Necrolysis of keratinocytes in the epidermis with superficial dermal neutrophilic and lymphocytic infiltrate	When caused by *S. aureus,* begins with fever, confusion, and fatigue. Erythema starts on the trunk, spreads to the extremities, and desquamates 1–2 weeks later. Sequelae can include Beau lines, nail shedding, and telogen effluvium
**Kawasaki disease** [18,19]	Mostly affects children below the age of five	Exact etiology is unknown but thought to be due to an infectious trigger	Diagnostic criteria: fever at least 5 days and 4 of the following: Bilateral conjunctival injection, oropharyngeal changes, cervical lymphadenopathy, edema/erythema/desquamation of hands/feet, and polymorphous rash	Dilation of small vessels in the papillary dermis with infiltration of CD4+ T cells and macrophages in the dermis and epidermis	If left untreated, can lead to coronary aneurysms and sudden death.
**Scarlet Fever** [20]	School-aged children and teenagers are most commonly affected	*Streptococcus pyogenes* producing streptococcal pyrogenic exotoxins types A, B and C, often after tonsillitis or pharyngitis	Preceded by sore throat, fever, headache, malaise, vomiting. Blanchable erythema of neck, chest, axillae. Papular, non-blanching rash that spares the palms and soles and is classically described as a “sandpaper” rash. Petechial streaks in folds (Pastia lines), flushing of cheeks	Spongiosis and parakeratosis with perivascular neutrophilic infiltrates	Rash usually develops 2–3 days after initial infection and can desquamate up to 2 weeks later. Patients can return to normal activity once afebrile for 24 h

**Table 3:** Differential diagnosis of bullous impetigo.

Disorder	Demographics	Triggers	Clinical Presentation	Histopathology	Clinical Course
**Bullous impetigo** [4,15]	Newborns and infants most commonly affected	Caused by exfoliative toxins produced by *S. aureus,* which cleave DSG1, resulting in acantholysis and bullae formation	Small vesicles that grow into tense bullae in well-demarcated clusters at the initial site of infection. Bullae rupture and leave behind a narrow rim of scale. Systemic involvement is rare. Nikolsky sign is negative, and culture of bullae or erosions is positive.	Loss of cell adhesion in the superficial epidermis (granular layer) resulting in a subcorneal blister; mixed dermal inflammatory infiltrate, crusting, epidermal hyperplasia, and lesional cocci in clusters.	Usually resolves within 3–6 weeks. However, high-risk patients may develop SSSS, due to dissemination of exfoliative toxin.
**Psoriasis** [29]	Can occur at any age, but has bimodal peak of 20–30 and 50–60 years of age	Cutaneous injury (koebnerization), infections (HIV, *Streptococcus*), stress, medications, hypocalcemia	Salmon pink plaques with silvery scale on extensor surfaces	Stratum corneum with alternating neutrophils and parakeratosis, regular acanthosis, tortuous blood vessels in dermal papillae	Chronic condition that can wax and wane in severity. Usually requires treatment for remission
**Linear IgA bullous dermatosis** [29]	Can occur in children and adults	Infections, medications, malignancies	Tense bullae often in herpetiform or annular arrangement (crown of jewels) overlying erythema	Subepidermal blister with neutrophils. Immunofluorescence shows linear IgA deposits along the dermoepidermal junction	Responds quickly to dapsone. If untreated, typically lasts several years then resolves on its own
**Contact dermatitis** [29]	Children and adults	Common allergens: poison ivy, nickel, fragrances, neomycin	Well-demarcated pink scaly patches and thin plaques, variable overlying vesicles, bullae, or crusting	Spongiotic dermatitis (spongiosis, lymphocyte exocytosis, parakeratosis), often with eosinophils and Langerhans cell vesicles	Rash persists until causative agent is removed. Responds to steroids (oral or topical).
**Superficial pustular folliculitis** [29]	Children and adults	Occluded areas, beard	Folliculocentric pustules and papules with collarettes of scale often on the head, neck, trunk, buttocks, legs	Suppurative folliculitis and mixed perifollicular infiltrate including neutrophils and lymphocytes	Lesions come and go; individual lesions will often spontaneously resolve. Response to topical and oral antibiotics.
**Pemphigus foliaceus** [29]	Usually seen in adults 45–65 years old	Autoantibodies targeting desmoglein 1. Medications (captopril, penicillamine). Possible environmental triggers can cause Fogo Selvagem, a form of pemphigus foliaceus endemic to Brazil	Erythematous “puff pastry-like” crusted erosions often in seborrheic distribution, with a positive Nikolsky sign and no mucosal involvement	Subcorneal split in granular layer with acantholysis and scattered eosinophils +/− neutrophils. DIF shows intercellular IgG and C3 deposition primarily in the upper half of epidermis	Treatment with rituximab achieves complete remission in 90% of patients within two years

## Data Availability

Not applicable.
